# "Giant R wave" electrocardiogram pattern during exercise treadmill test: A case report

**DOI:** 10.1186/1752-1947-5-304

**Published:** 2011-07-11

**Authors:** Ana Testa-Fernández, Ramón Rios-Vazquez, Juan Sieira-Rodríguez-Moret, Raúl Franco-Gutierrez, Carlos Peña-Gil, Ruth Pérez-Fernández, Victor Puebla-Rojo, Margarita Regueiro-Abel, Carlos Gonzalez-Juanatey

**Affiliations:** 1 Department of Cardiology, Hospital Lucus Augusti, San Cibrao s/n, Lugo E-27003, Spain

## Abstract

**Introduction:**

The exercise treadmill test is widely used in the evaluation of patients with suspected or known coronary artery disease. The typical ischemic response used to be ST-segment depression.

**Case presentation:**

We describe a case of a 51-year-old Caucasian man with an unusual ischemic response during the exercise treadmill test: a "giant R wave" electrocardiogram pattern as a manifestation of hyperacute ischemia that resolved with sublingual nitroglycerin. Coronary catheterization showed a severe stenosis in a proximal dominant circumflex coronary artery. We hypothesize that, in this case, the "giant R wave" pattern was related to severe hyperacute ischemia due to coronary spasm superimposed on the atherosclerotic lesion, which probably caused complete occlusion of the artery. The patient was successfully treated with coronary percutaneous revascularization.

**Conclusions:**

This is a dramatic and rare ischemic response during the exercise treadmill test, in which, a rapid administration of nitroglycerin can prevent life-threatening events.

## Introduction

A typical ischemic response during an exercise treadmill test (ETT) is ST-segment depression. ST-segment elevation is present in only about 3.5% of patients who undergo this test [1] and is more specific for indicating the site of the culprit lesion [2,3]. The "giant R wave syndrome" was first described by Prinzmetal *et al*. [4] in the context of variant angina, and it is characterized by the appearance of a giant R wave, loss of the S wave, and merging of the QRS complex with the ST segment, causing a monophasic QRS-ST complex in leads facing the ischemic territory. This pattern may be rarely observed following coronary artery occlusion in acute myocardial infarction (MI), following variant angina during an ETT (or spontaneously at rest), and after percutaneous transluminal coronary angioplasty or experimental coronary artery ligation [5]. We report a case of a patient who developed the giant R wave syndrome during an ETT.

## Case presentation

**Figure 1:**
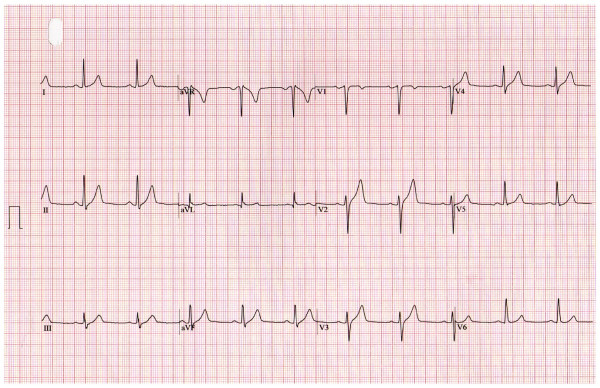
**Basal electrocardiogram**.

**Figure 2:**
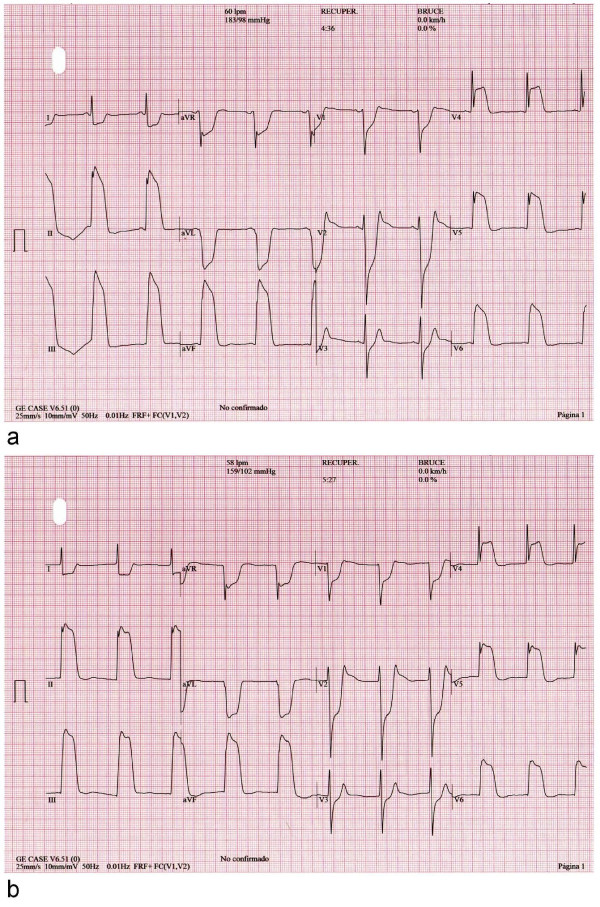
**(a) Electrocardiogram obtained in the recovery exercise treadmill test phase showing "giant R wave" pattern in inferolateral leads and nodal rhythm (b)**.

**Figure 3:**
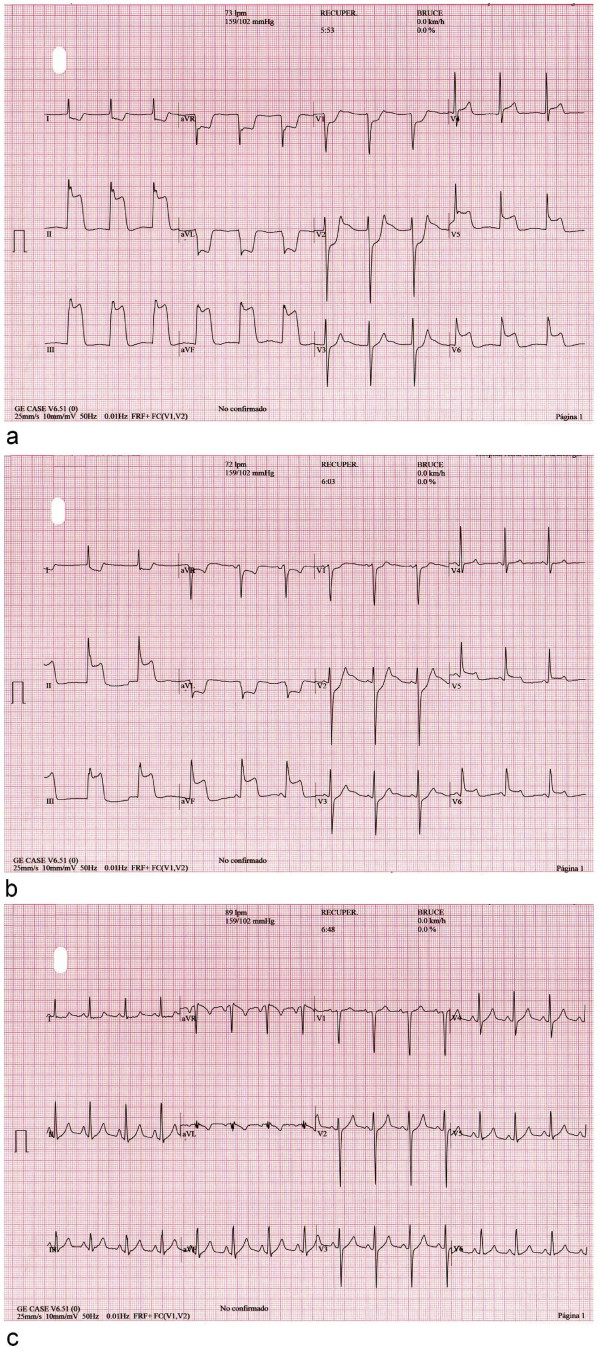
**(a) through (c) Evolution of electrocardiograms to normal status after nitroglycerin administration**.

**Figure 4:**
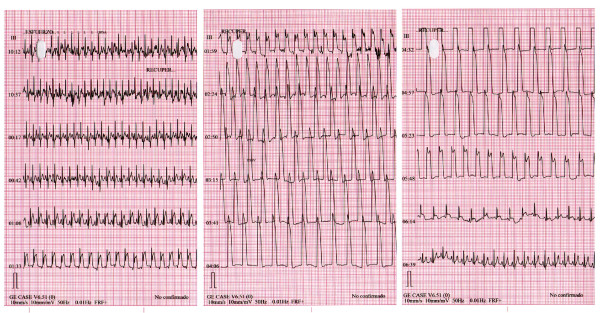
**Electrocardiographic record of "giant R wave" electrocardiogram pattern (lead III) during recovery phase and its normalization after nitroglycerin administration**.

**Figure 5:**
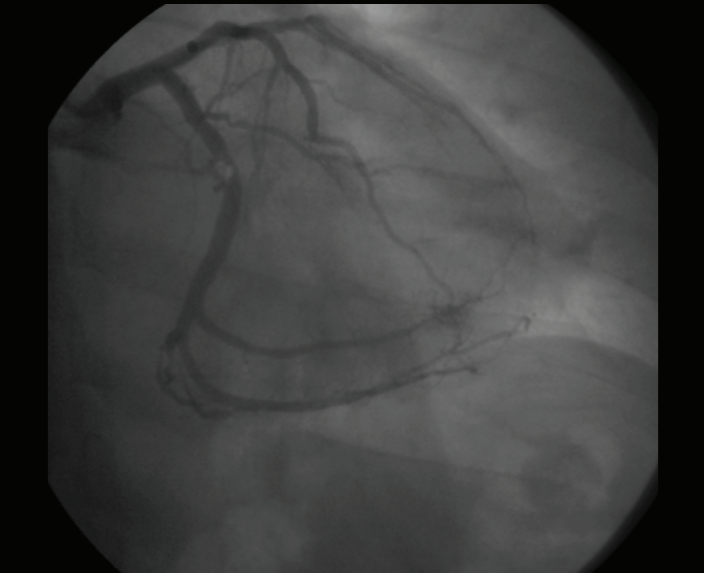
**Coronary angiography showing stenoses in mid-dominant circumflex coronary artery**.

**Figure 6:**
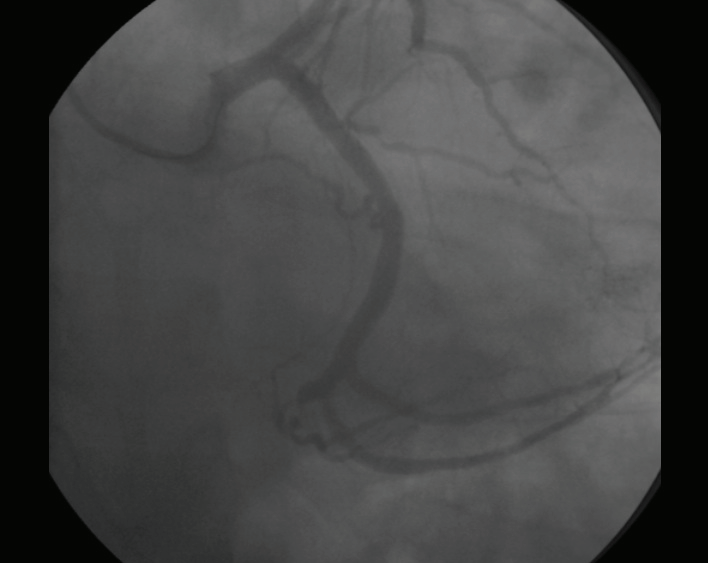
**Coronary angiography performed after percutaneous revascularization**.

A 51-year-old Caucasian man with the cardiovascular risk factors of hypertension and smoking was admitted to our hospital because of chest pain and syncope during exercise. He had experienced intermittent chest pain for one week prior to presentation. Upon admission, his blood pressure was 110/70 mmHg and his pulse rate was 70 beats/minute. His physical examination was unremarkable. His routine biochemistry laboratory parameters and full blood cell count were normal. A chest radiograph did not show indications of left-sided cardiac failure. A 12-lead electrocardiogram (ECG) obtained without chest pain in the patient showed normal sinus rhythm at 63 beats/minute without Q waves and with a normal QRS width (Figure 1). The echocardiogram showed a normal left ventricular ejection fraction without regional motion abnormalities. The patient was referred for exercise testing using a standard Bruce protocol. The test was stopped at minute 10 of the protocol because the patient was experiencing fatigue, had an increasing heart rate of 148 beats/minute (87% of maximal predicted heart rate), had maximal blood pressure of 183/98 mmHg, and had a workload of 12.8 metabolic equivalents without clinical symptoms or ECG abnormalities. At minute one of the recovery phase, the ECG showed a giant R wave pattern in the inferolateral leads, including an increase of R wave voltage rising to 34 mm, loss of S wave, and merging of the QRS complex with the ST segment, causing a monophasic QRS-ST complex (Figure 2a). A nodal rhythm was also observed (Figure 2b). Clinically, the patient experienced chest pain and dizziness. His blood pressure was 183/98 mmHg, and we administered sublingual nitroglycerin, and the patient's ECG gradually returned to normal status (Figure 3). In Figure 4, we show the evolution in lead III during the episode. The maximal level of troponin I measured after the ETT was 0.1 ng/mL (normal 0.0 ng/mL to 0.2 ng/mL). Emergency coronary arteriography showed single coronary vessel disease with severe stenosis in a mid-dominant circumflex coronary artery (Figure 5). The patient was successfully treated with coronary percutaneous revascularization (Figure 6), and, two days later, he was discharged without further symptoms.

## Discussion

The giant R wave syndrome was first described by Prinzmetal *et al*. [4] in the context of variant angina. Since then, similar ECG changes have been recorded in percutaneous transluminal coronary angioplasty, experimental coronary ligation, and in patients who had an acute MI (in this latter case, infrequent documentation exists in association with inferior MI, probably because of the smaller size of ischemic injury as compared to the anterior MI, resulting in less impressive ECG changes) [6]. The electrophysiological mechanism of the giant R waves in the setting of myocardial ischemia has been attributed by most investigators to the aberrant propagation of ventricular activation from the normal myocardium toward the ischemic area, in which there is a marked slowing of conduction velocity [6]. In variant angina, ST-segment elevation can be provoked by exercise, hyperventilation, or cold stimulation in about 30% of patients [7]. In our patient, the giant R wave pattern was provoked by exercise during ETT, which also showed nodal rhythm, and resolved with sublingual nitroglycerin administration. Coronary catheterization showed severe stenosis in a mid-dominant circumflex coronary artery, with an extensive territory at risk. We hypothesize that, in our patient, the giant R wave pattern was related to severe hyperacute ischemia due to a coronary spasm superimposed on the atherosclerotic lesion, which probably caused a complete occlusion of the artery. This severe ischemic response could explain the patient's prior symptoms due to cardiac rhythm and blood pressure abnormalities.

## Conclusions

In this case report, we describe a dramatic, rare ischemic response during ETT that was probably caused by superimposition of a coronary spasm in a severe stenosis of a mid-dominant circumflex coronary artery. Rapid administration of nitroglycerin is crucial because it can prevent life-threatening events.

## Consent

Written informed consent was obtained from the patient for publication of this case report and any accompanying images. A copy of the written consent is available for review by the Editor-in-Chief of this journal.

## Competing interests

The authors declare that they have no competing interests.

## Authors' contributions

AT performed the ETT and drafted the manuscript. CP helped in the management of the patient. RP obtained the echocardiograms. RR, VP, and MR were responsible for the management of the patient during his hospitalization. JS and RF performed the literature search. CG was a major contributor in writing the manuscript. All authors read and approved the final manuscript.
